# Differentially expressed lncRNAs and mRNAs identified by NGS analysis in colorectal cancer patients

**DOI:** 10.1002/cam4.1696

**Published:** 2018-07-23

**Authors:** Meng Li, Lian‐mei Zhao, Suo‐lin Li, Jing Li, Bo Gao, Fei‐fei Wang, Sheng‐pu Wang, Xu‐hua Hu, Jian Cao, Gui‐ying Wang

**Affiliations:** ^1^ Pediatric Surgery The Second Hospital of Hebei Medical University Shijiazhuang Hebei China; ^2^ The Second General Surgery Hebei Medical University Fourth Affiliated Hospital and Hebei Provincial Tumor Hospital Shijiazhuang Hebei China; ^3^ Research Center Hebei Medical University Fourth Affiliated Hospital and Hebei Provincial Tumor Hospital Shijiazhuang Hebei China

**Keywords:** colorectal cancer, enrichment analysis, lncRNAs, mRNA, next‐generation sequencing

## Abstract

Long noncoding RNAs (lncRNAs) play an important role in gene regulation, but their impact on the pathogenesis of colorectal cancer and the biological function of cancer cells is unclear. In this study, we used next‐generation sequencing to study the differences in the expression profiles of lncRNAs and mRNAs in colorectal cancer tissues. We analyzed the differentially expressed genes by Gene Ontology/Kyoto Encyclopedia of Genes and Genomes (GO/KEGG) enrichment and predicted new lncRNA functions. Our results revealed that compared with lncRNAs and mRNAs in nontumor colorectal tissues, 1019 lncRNAs (512 upregulated, 507 downregulated) and 3221 mRNAs (1606 upregulated, 1615 downregulated) were differentially expressed in tumor colorectal tissues (fold change >2 and *P* < 0.05). We validated some of these genes by qPCR. Furthermore, we identified some new lncRNAs differently expressed in colorectal cancer samples from patients in northern China. We confirmed the function of lncRNA‐FIRRE‐201 and SLCO4A1‐AS1‐202 in colorectal cancer cells to provide an experimental basis for studies on their roles in the occurrence and development of colorectal cancer and in the regulation of networks.

## 1 INTRODUCTION

Colorectal cancer (CRC) is currently the third most common malignancy and the second leading cause of cancer‐related death worldwide.^1^ Although there has been great progress in understanding the molecular mechanism involved and treatment for CRC in recent years, the overall survival rate of patients with CRC has not changed significantly.^2^ For malignant tumors, early detection, diagnosis, and treatment can greatly improve patient survival.^3^ It is difficult to identify tumors <1 cm with routine diagnostic methods, and the sensitivity and specificity of traditional tumor markers are not sufficient. CRC genesis involves multifactorial and complex steps that require the exploration of corresponding biomarkers.

Recently, long noncoding RNAs (lncRNAs) have emerged as a new field of study.^4^ lncRNAs, which are more than 200 nt in length and do not code for proteins,^5^ have recently attracted increasing research interest due to their important role in the regulation of multiple biological processes, including survival, proliferation, migration, and genomic stability.^6^ These regions of the genome have essential functions in normal organic development and diseases, including cancer.^4^ Moreover, there is increasing evidence that many lncRNAs play significant roles in the regulation of CRC.^7^ Next‐generation sequencing (NGS) technology was developed at the beginning of the current century as an alternative to Sanger sequencing. Its main advantage is that it significantly increases the throughput of sequences by simultaneously executing thousands of sequencing reactions.^8^

In this study, NGS technology was used to study the differential expression profiles of lncRNAs and mRNAs in CRC tissues. Our results showed large differences between tumor and normal colorectal tissues. Furthermore, the lncRNA‐mRNA network was used to analyze the Gene Ontology/Kyoto Encyclopedia of Genes and Genomes (GO/KEGG) gene and predict the function of the lncRNAs. We identified seven significantly aberrantly expressed lncRNAs (lncRNA‐FIRRE‐201, SLCO4A1‐AS1‐202, LINC02163‐201, and FEZF1‐AS1‐203 were upregulated, and SLC30A10‐201, PGM5‐AS1‐202, and ADIPOQ‐AS1‐201 were downregulated) and six differently expressed mRNAs (NM‐001062, NM‐002994, and NM‐001012964 were upregulated, and NM‐005182, NM‐002594, and NM‐001080400 were downregulated). These lncRNAs and mRNAs represent potential molecular diagnostic markers or novel therapeutic targets for patients with CRC.

## 2 MATERIALS AND METHODS

### 2.1 Tissue collection

Between 2014 and 2016, matched CRC and adjacent nontumor colorectal tissues were obtained from patients with CRC undergoing resection at Fourth Affiliated Hospital of Hebei Medical University and Hebei Provincial Tumor Hospital. None of the patients were treated by radiotherapy or chemotherapy before surgery. Immediately after the operation, the tissues were frozen in liquid nitrogen and stored at −80°C. All tumor samples, which contained more than 80% tumor tissue, were blindly confirmed by 2‐3 pathologists to reduce heterogeneity. Informed consent was obtained from patients, and the study was approved by the relevant hospital committee.

### 2.2 RNA extraction

Total RNA was extracted from tissues using TRIzol reagent (Invitrogen Life Technologies, Carlsbad, CA). RNA quantity was assessed with the Thermo ND‐1000 ultramicro nucleic acid protein meter (OD 260 nm; Thermo, Waltham, MA), using standard denaturing agarose gel electrophoresis to assess RNA integrity.

### 2.3 Next‐generation sequencing of lncRNAs and mRNAs and data

Ribosomal RNA was extracted using Epicentre Ribo‐Zero rRNA Removal Kit (Epicentre, Madison, WI). The RNA‐Seq library preparation was then carried out. First‐strand and second‐strand cDNA synthesis for fragmented RNA, with a length of ~ 200 bp, cohesive subconnections, and low cycle enrichment were performed following the instructions of the kit (New England Biolabs, Ipswich, MA) and according to Ultra^™^ RNA Library of Illumina. Ten pairs of qualified specimens were evaluated using Agilent 2200 TapeStation (Agilent Technologies, Santa Clara, CA). Finally, sequencing of the libraries was carried out using the Illumina HiSeq 3000 platform (Illumina, San Diego, CA) at Guangzhou RiboBio Co., Ltd. (Guangzhou, China).

### 2.4 Preprocessing of sequencing reads/quality control

I. With the options TRAIL‐ING:20, MINLEN:235, and CROP:235, the Trimmomatic tool (V 0.36) was used to process the original fastq sequence to remove sequences of <20 nt of the phred mass fraction and to achieve a unified sequence length for downstream clustering processes. II. FastQC software (http://www.bioinformatics.babraham.ac.uk/projects/fastqc/) was used to check the read quality of the sequences. Trimmomatic was used to remove and trim reads. The sequencing reads were trimmed from the ends (base quality less than Q20) and filtered by length (<25). The sequence data were mapped to human reference genome hg19 using TopHat v2.0.20; gfold v1.1.2 was applied to calculate the number of reads mapped to each gene. Differential expression was assessed by DESeq (http://www.bioconductor.org/packages/release/bioc/html/DESeq.html) using TPM (Transcripts Per Million), which is the recommended and most common method to estimate the level of gene expression. Differentially expressed genes were selected based on fold change >2 and adjusted *P*‐value<0.05.

### 2.5 Identification of new lncRNAs

Raw data were first filtered to remove low‐quality reads, as based on read mapping to the reference genome, and StringTie was employed to assemble clean data that had been repeatedly tested. The combined transcripts were annotated using the GFFCompare program. Screening putative lncRNAs used unknown transcripts. We identify lncRNA according to the following conditions: 1 Filter the genes and lncRNA known to the database; 2 RNA with a length greater than 200nt; 3 Predictive open reading Frame (ORF) <300nt; 4 Filtering RNA with PFAM domains; 5 Filtering RNA with coding potential. Through the above steps, the new transcripts with lncRNA characteristics were screened, and finally recognized coding potential score less than 1 as a new lncRNA by the CPC software (http://cpc.cbi.pku.edu.cn/). Raw data were first filtered to remove low‐quality reads. StringTie was then used to assemble retested clean data based on reads mapped to the reference genome. Assembled transcripts were provided exegesis using the GFFCompare program, and unknown transcripts were screened for presumed lncRNAs. Filtering of putative protein‐encoding RNA was achieved using the minimum length and exon number threshold. lncRNA candidates were selected via transcript lengths greater than 200nt and an ORF shorter than 300nt, and CPC/CNCI/PFAM was employed to further screen coding and noncoding genes.

### 2.6 Hierarchical clustering analysis

Statistically significant genes were obtained according to *P* < 0.05 and |log2FoldChange|>3 using DESeq software. Hierarchical clustering analysis was carried out using the R language package, according to the TPM values of differential genes in different groups. The TPM value was the expression level, and the expression patterns were clustered into the same or similar genes by hierarchical clustering analysis, using different colors to represent different subregional information to determine the clustering pattern control model in different experimental conditions.

### 2.7 GO analysis

Gene Ontology analysis, which provides label classification of gene function and gene product attributes (http://www.geneontology.org), was performed with KOBAS3.0 software. GO analysis was performed for each gene to assess functional annotations, and a series of specific gene functions was calculated through hypergeometric distribution. GO functional annotations provide differential gene expression molecular function, biological process, and cellular component and provide genes and gene function research background knowledge for classification and labeling. GO analysis of expression of different genes (|log2FoldChange|>1 and *q*‐value<0.05) uses the hypergeometric distribution method to calculate *P*‐values and obtain high‐frequency GO category information and significant cases, with *P* < 0.05 as the significant threshold of statistical significance.

### 2.8 KEGG analysis

Biological pathway analysis based on the biology of KEGG pathways with KOBAS3.0 software (http://www.genome.jp/kegg), from the viewpoint of a complex regulatory network, was used for enrichment analysis to study biological function. We analyzed differentially expressed genes in biological pathways to determine whether functional variation affected the degree and pattern of biological pathways. Differentially expressed genes revealed by the KEGG pathway provide signal transduction and disease pathway annotation information. Fisher's exact test was used, with *P* < 0.05 as the significance threshold.

### 2.9 Coexpression network of differentially expressed lncRNAs‐mRNAs

The lncRNA‐mRNA transcript coexpression network was constructed to examine the potential functions of differentially expressed lncRNAs and lncRNA‐mRNA interactions. The coexpression network was constructed by calculating the Pearson correlation coefficients and *P*‐values of filtered transcripts using *P* < 0.05 and COR>0.85. We constructed seven coexpression networks, which were illustrated using Cytoscape software (http://www.cytoscape.org/).

### 2.10 qRT‐PCR analyses

For quantitative real‐time PCR (qRT‐PCR), the amount of total RNA used was 1 μg. RNA was reverse‐transcribed to complementary DNA (cDNA) using a Transcriptor First Strand cDNA Synthesis Kit (Roche, Mannheim, Germany). Real‐time PCR analyses were performed with SYBR Green (TransGen Biotech, Beijing, China), using the ABI Prism 7500 system (Applied Biosystems, USA), under the following conditions: (a) 94°C for 30 seconds; (b) 40 cycles of 94°C for 5 seconds and 60°C for 30 seconds; and (c) 95°C for 1 minutes, 55°C for 30 seconds, and then 95°C for 30 seconds. The relative fold change was calculated using the 2^−∆∆Ct^ method normalized to GAPDH. Each sample was analyzed in triplicate. The primers are described in Table S1.

### 2.11 Cell lines and culture conditions

The human CRC cell lines HCT116 and SW480 were obtained from GeneChem Company. All cell lines were grown and maintained in RPMI‐1640 medium (GIBCO) supplemented with 10% fetal bovine serum (FBS; BI), 100 U/mL penicillin, and 100 mg/mL streptomycin (Solarbio, Beijing, China) in an incubator at 37°C with 5% CO_2_.

### 2.12 RNA interference by siRNA

Small interfering RNA (siRNA) and nonspecific control siRNA were synthesized (Invitrogen Biotech, Shanghai, China). Each sample was analyzed in triplicate and transfected into HCT116 and SW480 cells using Lipofectamine 2000 (Invitrogen, USA) according to the manufacturer's instructions. The siRNA sequences are displayed in Table S2. Every test was carried out in triplicate, with three technical replicates.

### 2.13 Cell proliferation and colony formation assay

The MTS assay was used to assess cell proliferation. At 24 hours after cell transfection, 2000 transfected cells were plated in each well of a 96‐well plate with six replicate wells and assessed every 24 hours following the manufacturer's instructions. Every 24 hours, 20 μL MTS (Solarbio, Beijing, China) was added, and measurements were performed using an enzyme‐labeled instrument (Anthos, Austria). Every test was carried out in triplicate, with six technical replicates. For the colony formation assay, 2000 cells were plated into each well of a 6‐well plate after transfection for 24 hours and maintained in media containing 10% FBS for approximately 1 week; the medium was replaced every 3 days. The colonies were fixed with methanol and stained with 0.1% crystal violet; the number of colonies (more than 0.3 mm) was then counted. Every SW480 test was carried out in triplicate, and it was repeated four times for HCT116.

### 2.14 Cell migration and invasion assay

Transwell chambers (Costar, Corning, NY) with a polycarbonic membrane (6.5 mm diameter, 8 μm pore size) were used to determine the migration and invasion of SW480 and HCT116 cells in vitro after transfection for 24 hours. The transfected cells were resuspended in the upper chamber at a density of 12 × 10^5 ^cells/mL in 200 μL of serum‐free medium for the migration assay. Next, 600 μL of RPMI‐1640 supplemented with 20% FBS was added to the lower chamber. After incubation (HCT116 for 24 hours; SW480 for 48 hours), the cells on the upper membrane surface were mechanically removed. Cells that had migrated or invaded to the underside of the membrane were fixed with methanol and stained with crystal violet (Solarbio, Beijing, China). Stained cells were counted in five randomly chosen fields, and the average number was calculated at 200x magnification using Image‐Pro Express software and a TS100 inverted microscope (Nikon, Japan). The transwell membrane was coated with 15 μL of Matrigel solution (500 ng/μL; BD, Franklin Lakes, NJ) and incubated at 37°C for 4 hours for the invasion assay. The remaining steps were similar to the migration assay. Every HCT116 test was carried out in triplicate, and it was repeated four times for SW480.

### 2.15 Wound‐healing assay

Cells were seeded on a 6‐well plate in medium containing 10% FBS after transfection for 24 hours. The confluent monolayer was scratched with a plastic tip; PBS was used to remove cell debris, and the samples were maintained in a 37°C incubator with 5% CO_2_ for 24 (HCT116) or 48 (SW480) h. Measurement of wound closure was performed in five random fields at 100x magnification using Image‐Pro Express software and a TS100 inverted microscope (Nikon, Japan). Wound healing was measured and evaluated with SPSS statistics. Every test was carried out in triplicate.

### 2.16 Statistical analyses

**Figure 1:**
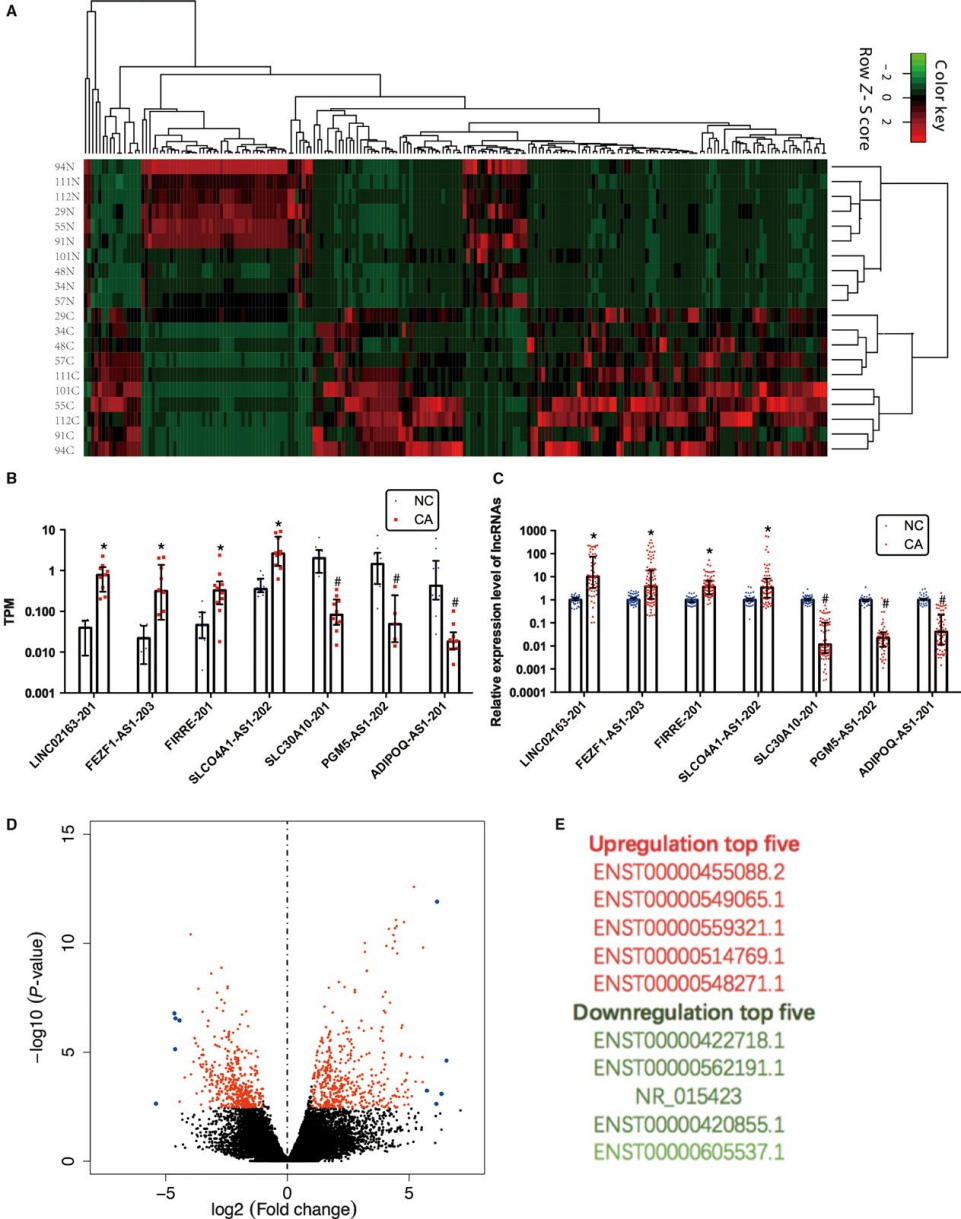
Differential expression of lncRNAs in colorectal cancer. (A) Heat map and hierarchical clustering (|log2FoldChange|>3 and *q*‐value <0.05) are presented to show the variation in lncRNAs between paired colorectal cancer and adjacent nontumor colorectal tissues. Red indicates high expression, and green indicates low relative expression. (B and C) Differential expression results of sampling lncRNAs from next‐generation sequencing analysis (B) and validating with qPCR (C) (*Expressed upregulated genes, #Expressed downregulated genes). Nonparametric tests were used. (D) The volcano plot (|log2FoldChange|>1 and *q*‐value <0.05) can reflect the gene expression difference. (E) The top five upregulated and deregulated lncRNAs between paired CRC and adjacent nontumor colorectal tissues (fold change > 2 and *P* < 0.05)

**Figure 2:**
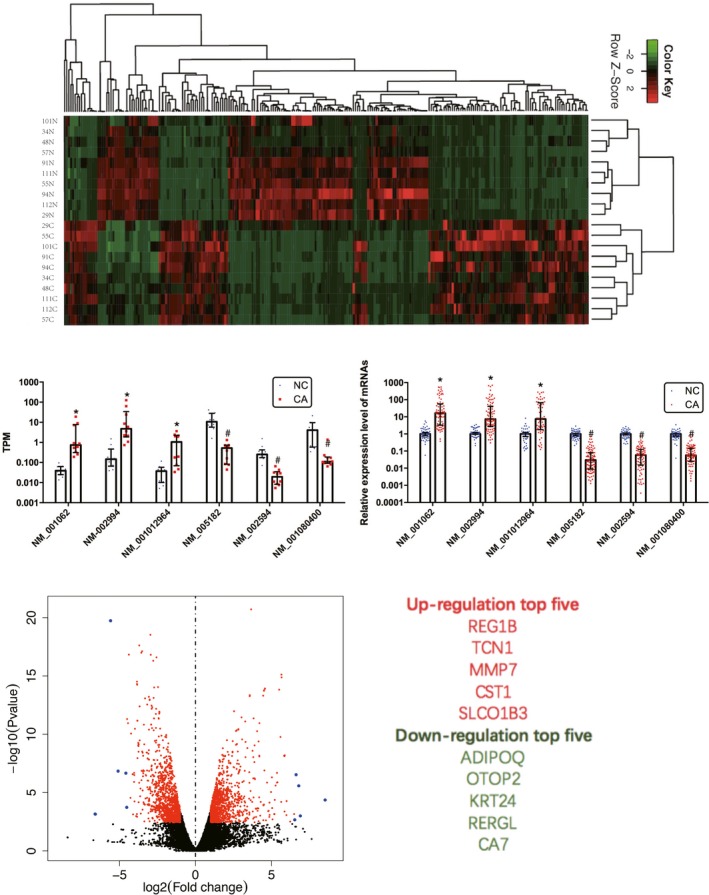
Differential expression of mRNAs in colorectal cancer. (A) Heat map and hierarchical clustering (|log2FoldChange|>3 and *q*‐value <0.05) are presented to show the variation in mRNAs between paired colorectal cancer and adjacent nontumor colorectal tissues. (B and C) Differential expression results of sampling mRNAs from the next‐generation sequencing analysis (B) and validating with qPCR (C). (*Expressed upregulated genes, #Expressed downregulated genes) Nonparametric tests were used. (D) The volcano plot (|log2FoldChange|>1 and *q*‐value <0.05) can reflect the gene expression differences. (E) The top five upregulated and deregulated mRNAs between paired CRC and adjacent nontumor colorectal tissues (fold change > 2 and *P* < 0.05)

Data were analyzed using SPSS 23.0 software (SPSS, Chicago, IL), and the cell function experiments were presented as the mean ± standard deviation (SD), with one‐way ANOVA and Bonferroni test for the comparison of normally distributed data. One‐way repeated‐measures ANOVA was used for MTS. Nonparametric tests were used for comparing and validating sequencing and qPCR of matched CRC and adjacent nontumor colorectal tissues, including differential expression results of sampling lncRNAs and mRNAs from next‐generation sequencing analysis (Figures 1B and 2B), and validating with qPCR (Figures 1C and 2C). The bar stood for 25% and 75% median with interquartile range. Differences were considered to be significant when *P* < 0.05.

## 3 RESULTS

### 3.1 Differential expression of lncRNAs in colorectal cancer

Next‐generation sequencing was used to evaluate the lncRNA expression in paired CRC and adjacent nontumor colorectal tissues. To identify differentially expressed lncRNAs, genes with more than 2‐fold expression changes (*P* < 0.05) were selected. Hierarchical clustering indicated the differentially expressed lncRNAs (|log2FoldChange|>3 and *q*‐value<0.05) (Figure 1A) among paired CRC and adjacent nontumor colorectal tissues to determine the similarity in gene expression. The volcano plot (|log2FoldChange|>1 and *q*‐value <0.05) in Figure 1D shows the overall gene expression levels. The top five upregulated and deregulated lncRNAs are listed in Figure 1E.

Compared with the lncRNAs in nontumor colorectal tissues, 1019 lncRNAs (512 upregulated, 507 downregulated) were differentially expressed in CRC tissues (fold change >2 and *P* < 0.05). To validate our sequencing data, 7 lncRNAs (LINC02163‐201, FEZF1‐AS1‐203, FIRRE‐201, SLCO4A1‐AS1‐202, SLC30A10‐201, PGM5‐AS1‐202, and ADIPOQ‐AS1‐201) were randomly selected for detection by quantitative real‐time PCR. In this validation, seven lncRNAs had the same expression pattern as the sequencing analysis (Figure 1B,C).

### 3.2 Differential expression of mRNAs in colorectal cancer

Sequencing analysis was performed to examine the expression profile of mRNA in the paired CRC and adjacent nontumor colorectal tissues. Hierarchical clustering (|log2FoldChange|>3 and *q*‐value <0.05) indicated the differentially expressed mRNAs (Figure 2A). The volcano (|log2FoldChange|>1 and *q*‐value <0.05) (Figure 2D) indicates differences in gene expression. A total of 3221 mRNAs (1606 upregulated, 1615 downregulated) were differentially expressed in CRC tissues (fold change >2 and *P* < 0.05). The top five upregulated and deregulated mRNAs are listed in Figure 2E. To validate our sequencing data, six mRNAs (NM_001062, NM_002994, NM_001012964, NM_005182, NM_002594, and NM_001080400) were randomly selected for detection by quantitative real‐time PCR. The qPCR results showed that compared with the paired tissues, the validated mRNAs had the same expression trend as the results obtained by the NGS analysis (Figure 2B,C).

The sequencing data have been deposited in GEO (GSE 104836; see https://www.ncbi.nlm.nih.gov/geo/query/acc.cgi?acc=GSE104836).

### 3.3 New target gene prediction for the differentially expressed lncRNAs

**Figure 3:**
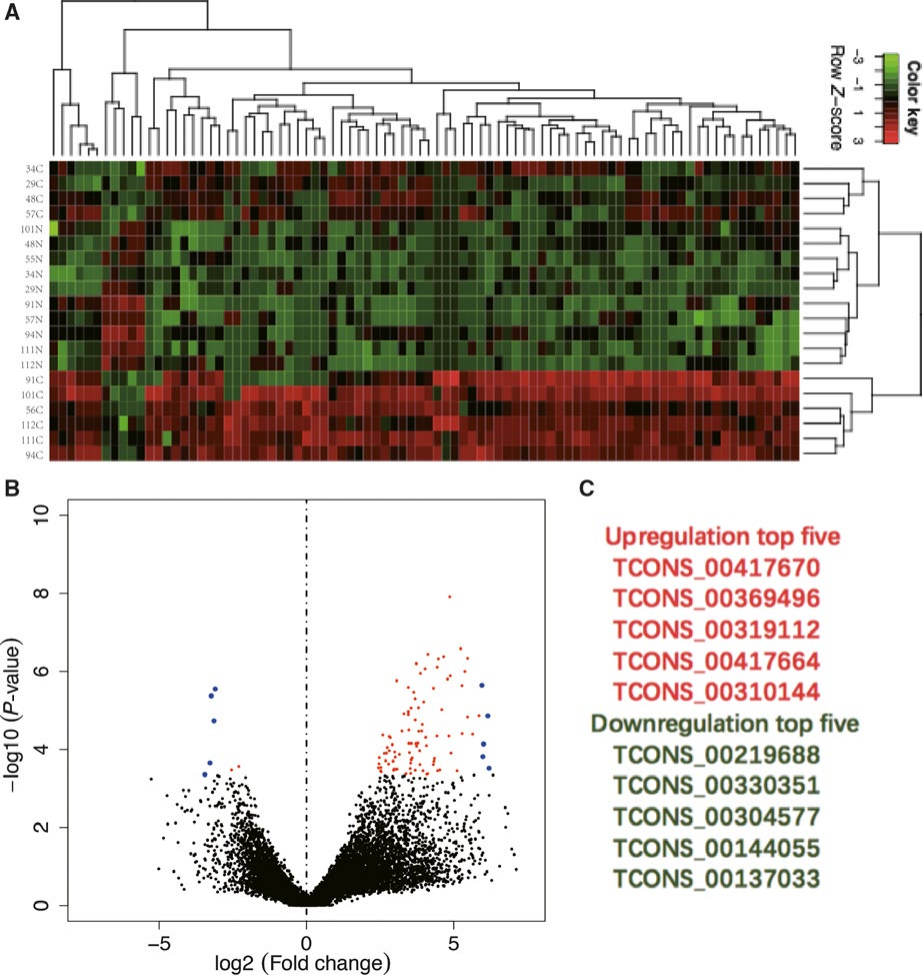
The new target gene prediction for the differentially expressed lncRNAs. A, Heat map and hierarchical clustering (|log2FoldChange|>3 and *q*‐value <0.05) are presented to show the variation in the new target genes between paired colorectal cancer and adjacent nontumor colorectal tissues. B, The volcano plot (|log2FoldChange|>1 and *q*‐value <0.05) can reflect the gene expression difference. C, The top five upregulated and deregulated new lncRNAs between paired CRC and adjacent nontumor colorectal tissues (fold change >2 and *P* < 0.05)

Based on known genetic models, new transcribed regions were predicted using sequencing data from all samples. The software package Cufflinks was used to assemble the transcriptase to identify new transcribed regions (FM >1 and overlaps with known genes before and after 200 bp) and to analyze the expression levels of the newly transcribed regions. Target gene prediction for the differentially expressed lncRNAs was represented by hierarchical clustering (|log2FoldChange|>3 and *q*‐value <0.05) (Figure 3A) and the volcano plot (|log2FoldChange|>1 and *q*‐value <0.05) (Figure 3B). We identified 109 new lncRNAs in this assay; 102 were upregulated and seven were downregulated. The top five upregulated and deregulated new lncRNAs are listed in Figure 3C.

### 3.4 GO and KEGG analysis

**Figure 4:**
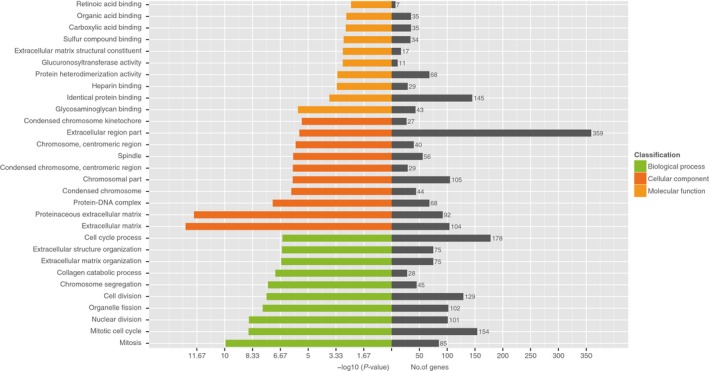
GO analysis of differentially expressed mRNAs according to molecular function, biological process, and cellular component. The differentially expressed genes were analyzed by GO, the *P* < 0.05 of GO annotation was regarded as the significant threshold, and the analysis results were obtained

Genes with more than 2‐fold change were analyzed by GO analysis. Those genes upregulated and downregulated in paired CRC and adjacent nontumor colorectal tissues were found to be involved in biological process, cellular component, and molecular function. The upregulated lncRNAs were primarily involved in “extracellular matrix,” “proteinaceous extracellular matrix,” “mitosis,” and “glycosaminoglycan binding,” and the lncRNAs were significantly enriched in “extracellular region part,” “cell cycle process,” and “mitotic cell cycle” (Figure 4).

**Figure 5:**
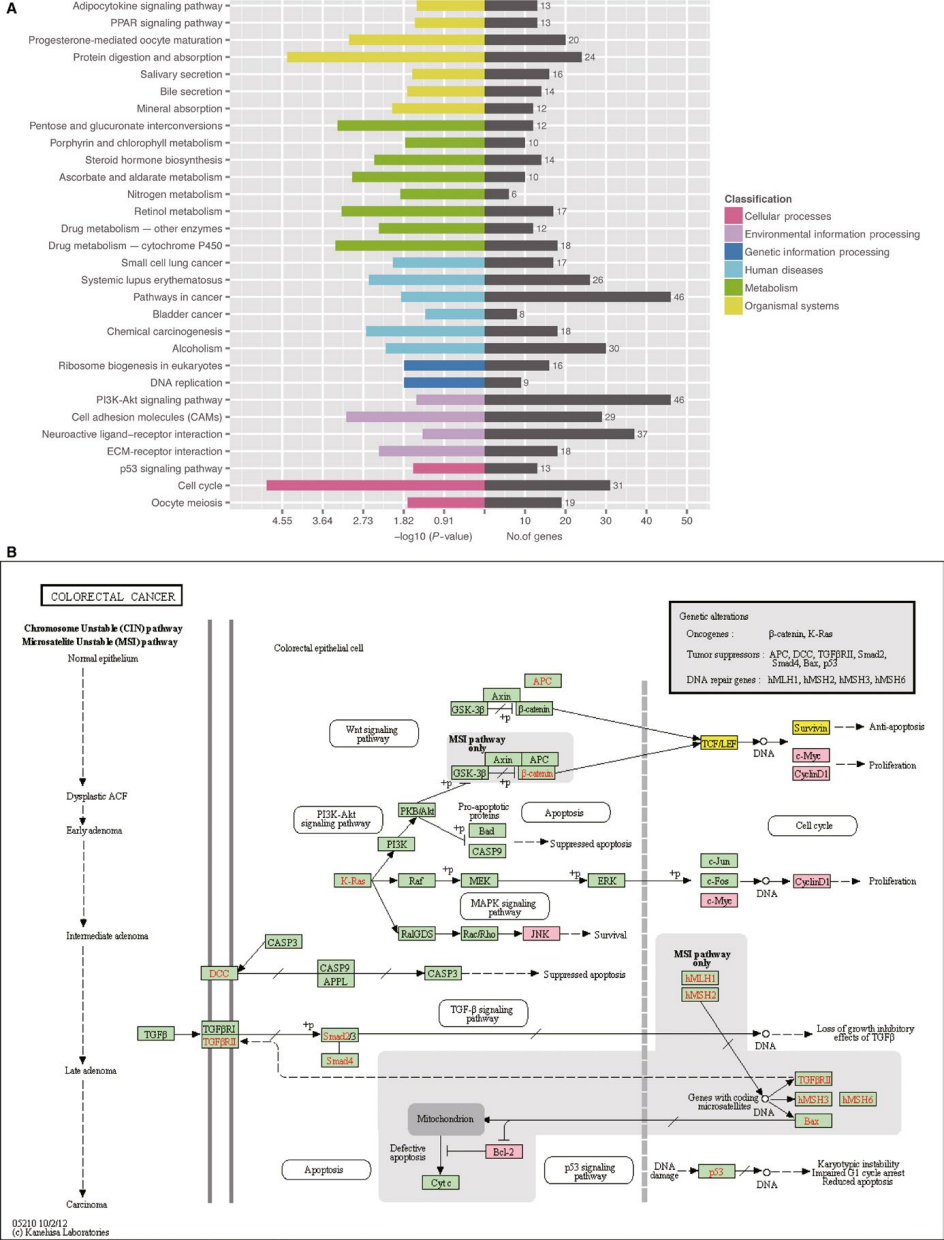
KEGG. A, Enrichment statistics of KEGG pathway in a sample‐classification map. B, The colorectal cancer signaling pathway. The pink background box indicates a differential upregulated gene, and yellow indicates a differential downregulated gene

Kyoto Encyclopedia of Genes and Genomes pathway analysis was used to investigate the biological pathways of the differentially expressed lncRNAs (Figure 5A). The upregulated lncRNAs were significantly involved in “cell cycle,” “protein digestion and absorption,” “Drug metabolism‐cytochrome P450,” “Pentose and glucuronate interconversions,” “Retinol metabolism,” “cell adhesion molecules,” “Progesterone‐mediated oocyte maturation,” “ascorbate and aldarate metabolism,” and “Chemical carcinogenesis.” The composition and pathway relationships of mutant molecules in signaling pathway were also investigated. Annotation of the KEGG pathway provides annotation information for signal transduction and disease pathways for differentially expressed genes, thus providing background information on gene pathways and functional studies. Based on the above analysis, we obtained many signal pathway maps. We chose to focus on the biological pathways for colon cancer (Figure 5B). We conducted a signal pathway search for colon cancer according to the sequencing data, including the Wnt signaling pathway, the PI3K‐Akt signaling pathway, the TGF‐β signaling pathway, the MAPK signaling pathway, and the p53 signaling pathway.

### 3.5 Construction of lncRNA/mRNA coexpression network

**Figure 6:**
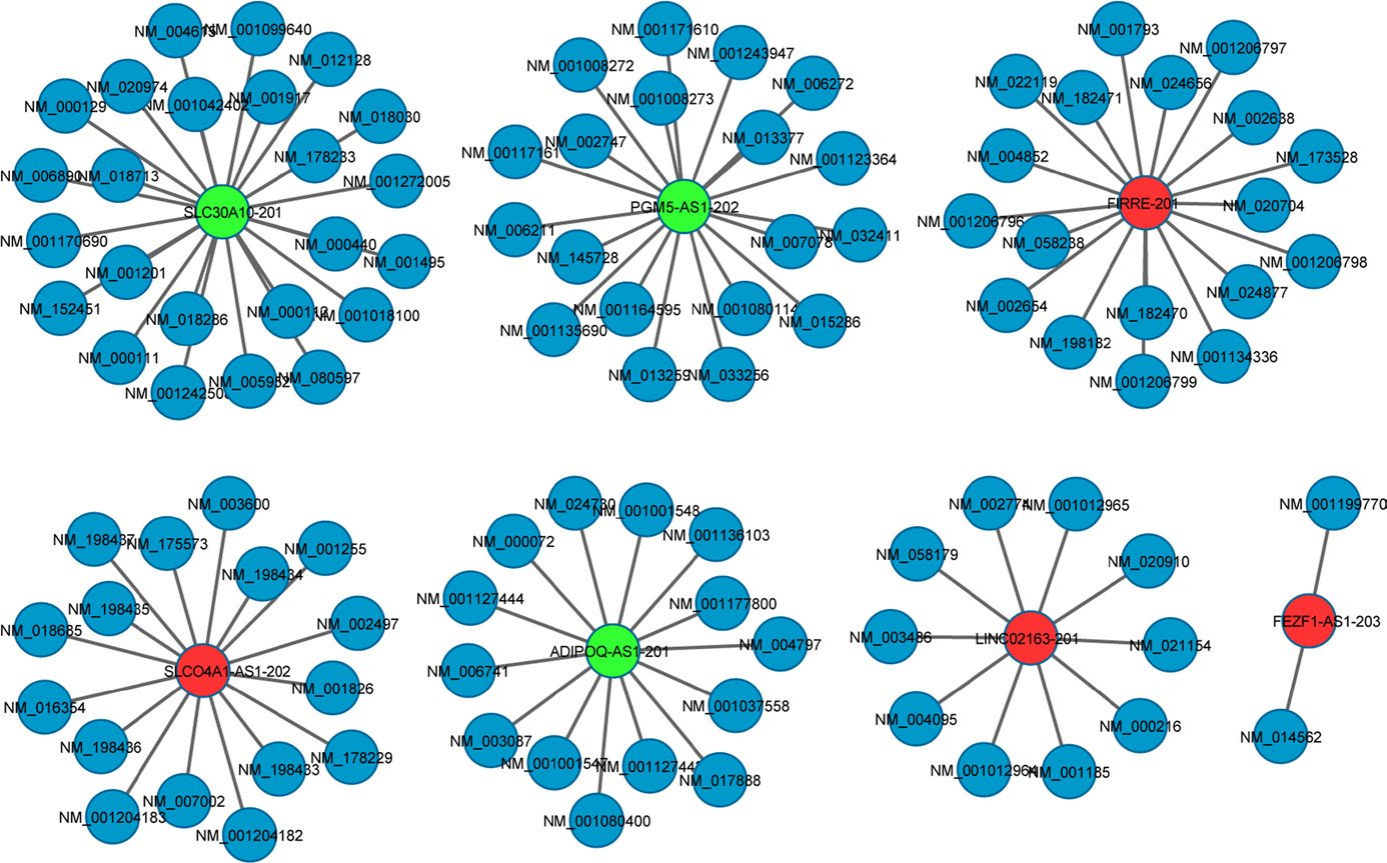
Coexpression networks of lncRNAs and relative mRNAs. A total of 103 mRNAs interacted with seven lncRNAs. Genes colored in red are upregulated lncRNAs, genes colored in green are downregulated lncRNAs, and genes colored in blue are mRNA

The correlation coefficient was set to 0.85, the lncRNA/mRNA coexpression network (Figure 6) was formulated, and the potential mRNAs associated with lncRNA/mRNA were predicted. We constructed coexpression networks to identify genes that are coexpressed with lncRNA as potential target genes for lncRNA. We drew the network of seven lncRNAs (LINC02163‐201, FEZF1‐AS1‐203, FIRRE‐201, SLCO4A1‐AS1‐202, SLC30A10‐201, PGM5‐AS1‐202, and ADIPOQ‐AS1‐201) using the Cytoscape program. The network indicated that each lncRNA correlated with two to more than 20 mRNAs.

### 3.6 Experimental validation of cell function

**Figure 7:**
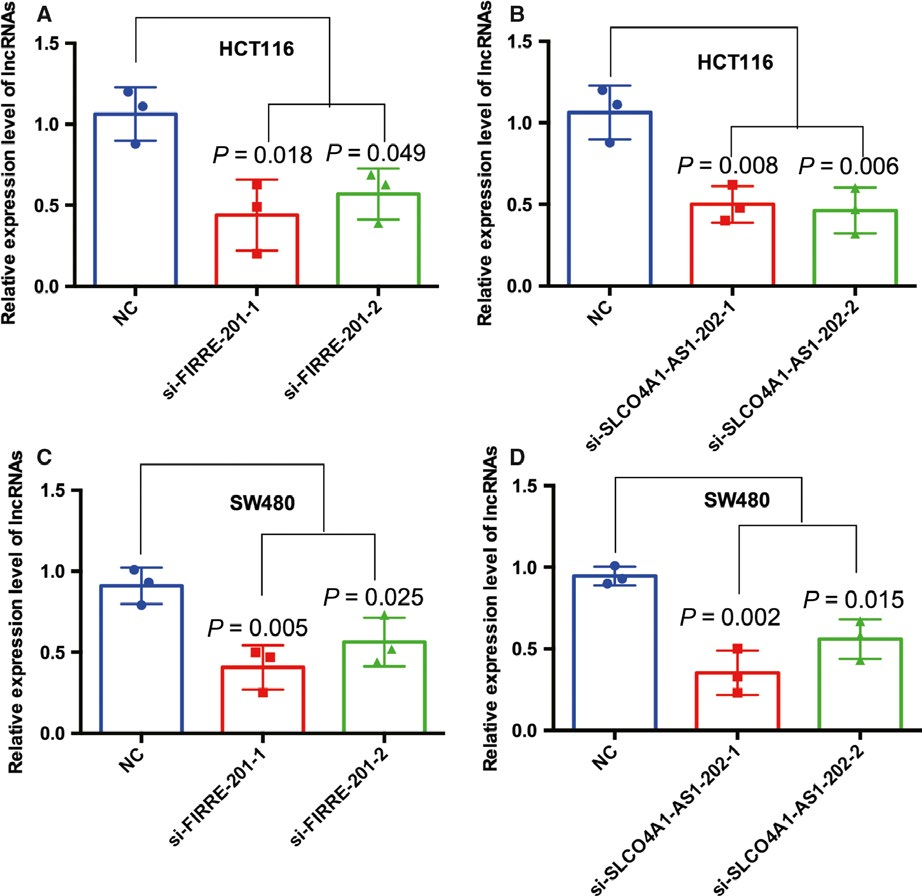
The expression levels of mRNA in CRC HCT116 and SW480 cells after transfection with siRNA were examined by qPCR. A, HCT116 were transfected with si‐FIRRE‐201‐1 and si‐FIRRE‐201‐2, and the expression levels of mRNA were lower. B, HCT116 were transfected with si‐SLCO4A1‐AS1‐202‐1 and si‐SLCO4A1‐AS1‐202‐2, and the expression levels of mRNA were lower. C, SW480 were transfected with si‐FIRRE‐201‐1 and si‐FIRRE‐201‐2, and the expression levels of mRNA were lower. D, SW480 were transfected with si‐SLCO4A1‐AS1‐202‐1 and si‐SLCO4A1‐AS1‐202‐2, and the expression levels of mRNA were lower. Every test was carried out in triplicate, with three technical replicates. Data are presented as mean ± SD

**Figure 8:**
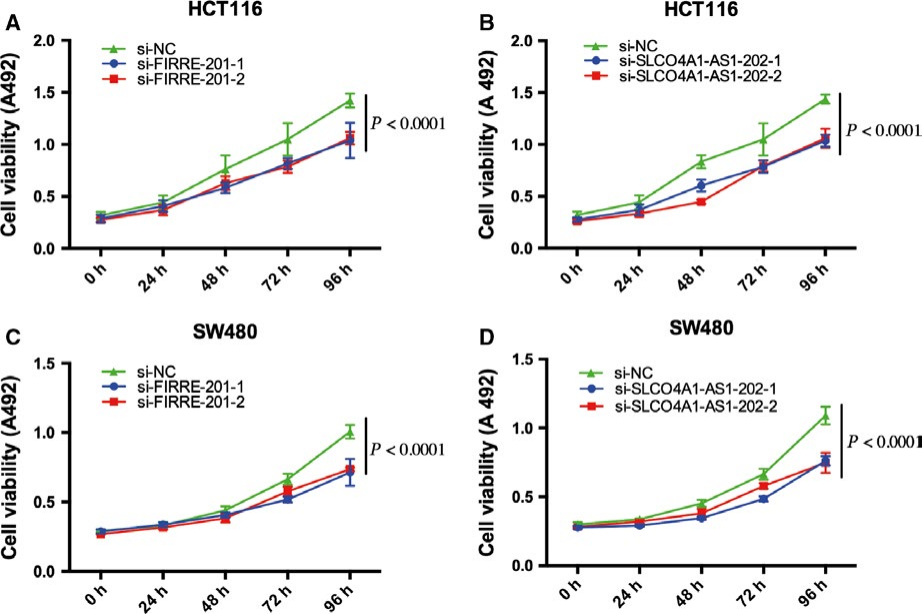
Knocking down lncRNA‐FIRRE‐201 or lncRNA‐SLCO4A1‐AS1‐202 reduced CRC cell growth. Proliferation of CRC cells transfected with siRNA against FIRRE‐201‐1, FIRRE‐201‐2 (A, C), and siRNA control was assessed by the MTS assay. Proliferation of CRC cells transfected with siRNA against SLCO4A1‐AS1‐202‐1, SLCO4A1‐AS1‐202‐2 (B, D), and siRNA control was assessed by the MTS assay. *P*‐values are obtained based on one‐way repeated‐measures ANOVA. Every test was carried out in triplicate, with 6 technical replicates

**Figure 9:**
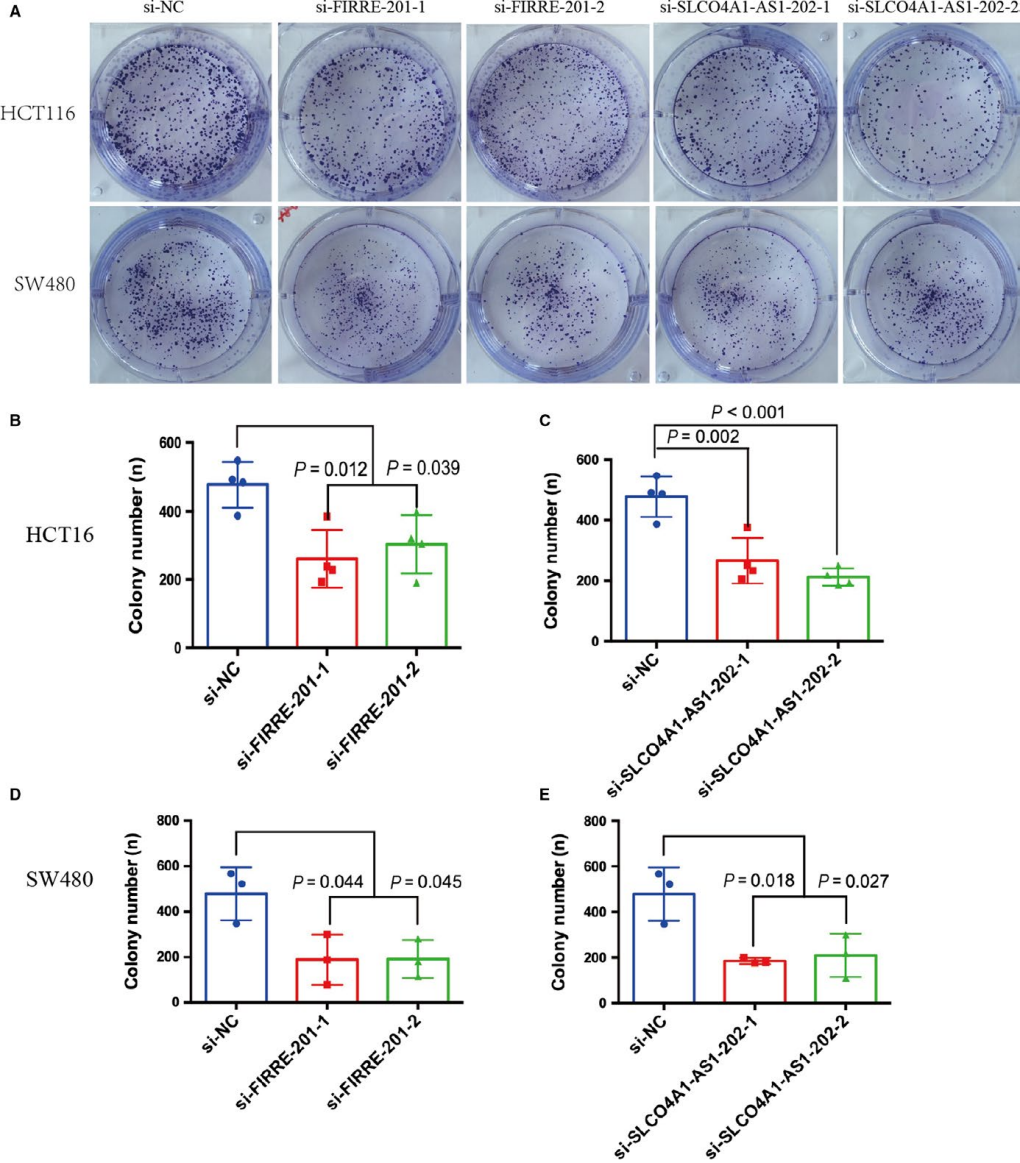
Knocking down lncRNA‐FIRRE‐201 or lncRNA‐SLCO4A1‐AS1‐202 reduced CRC colony formation. HCT116 or SW480 were transfected with siRNA against FIRRE‐201 (B, D), SLCO4A1‐AS1‐202 (C, E), or siRNA control for 24 h, and then, 2000 cells from each group were plated in 6‐well plates for 7 d. The number of colonies was calculated and plotted on a histogram (A). *P*‐values are obtained based on one‐way ANOVA. Every SW480 test was carried out in triplicate, and it was repeated four times for HCT116

**Figure 10:**
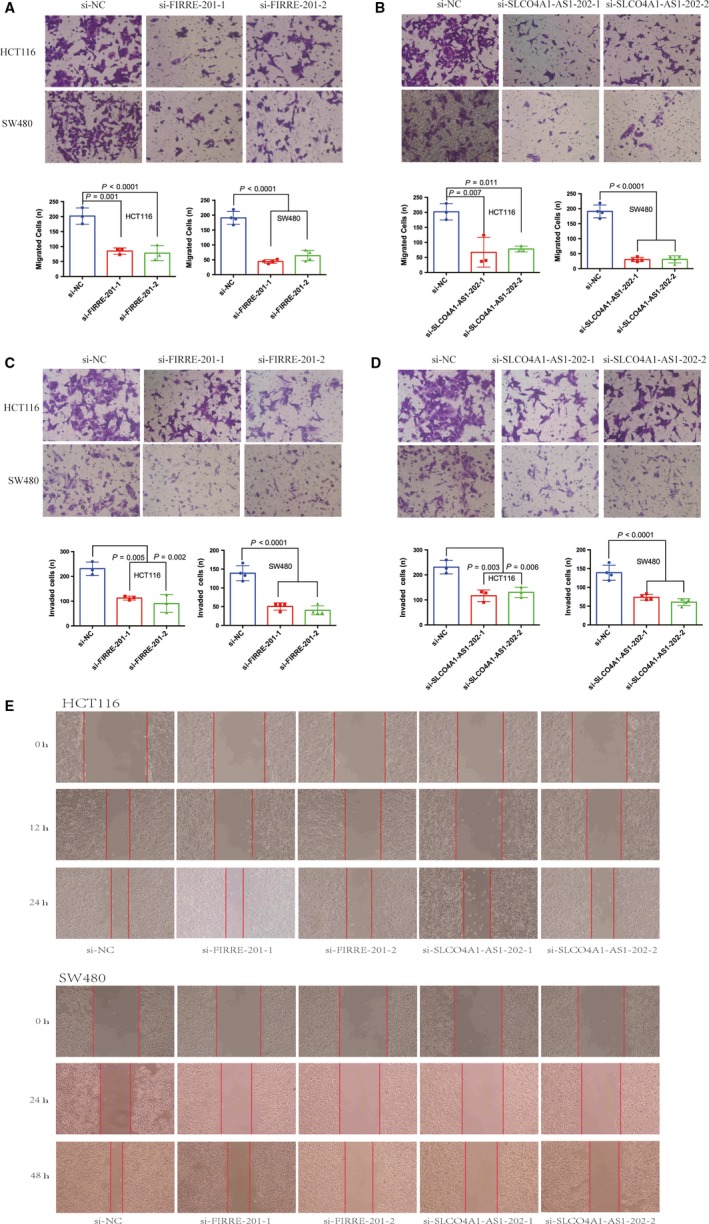
Knocking down lncRNA‐FIRRE‐201 or lncRNA‐SLCO4A1‐AS1‐202 reduced CRC migration and invasion. Knockdown of FIRRE‐201 (A, C) and SLCO4A1‐AS1‐202 (B, D) on the migration and invasion of CRC cells was investigated using transwell and Matrigel assays, respectively, showing the average counts from five random microscopic fields. Every HCT116 test was carried out in triplicate, and it was repeated four times for SW480. *P*‐values are obtained based on one‐way ANOVA. Data are presented as mean ± SD. The effect of CRC cells transfected with siRNA against FIRRE‐201, SLCO4A1‐AS1‐202, and siRNA control on cellular mobility was investigated using a wound‐healing assay (E). Data are presented as mean ± SD. Every test was carried out in triplicate

We chose two differently expressed lncRNAs, FIRRE‐201 and SLCO4A1‐AS1‐202, to implement cell function tests and found that the two lncRNAs positively modulate CRC cellular malignant phenotypes. The expression levels of mRNA in HCT116 and SW480 cells after transfection with siRNA were examined by real‐time fluorescent quantitative PCR (Figure 7). Knockdown of FIRRE‐201 and SLCO4A1‐AS1‐202 reduced cell proliferation (Figure 8), colony formation (Figure 9), and migration and invasion (Figure 10). All experiments were conducted at least 3 times, and the data were statistically analyzed by the normality test at first, then one‐way ANOVA to compare the differences between the three groups, and finally, Bonferroni's test for multiple comparisons (Figures 7, 9, and 10). One‐way repeated‐measures ANOVA was used for MTS (Figure 8). In vitro biological/experimental samples in replicates are marked on figure legends.

## 4 DISCUSSION

Dysregulation of long noncoding RNAs has been found in many human cancers, including CRC, which is the third most prevalent cancer worldwide and has a poor prognosis. In addition, robust evidence has indicated that lncRNAs can affect tumor suppressor genes or oncogenes.^9^ However, lncRNA‐mRNA expression profiles from northern China have not been generated. We profiled the expression of lncRNAs and mRNAs in CRC tissues by NGS and identified lncRNAs and mRNAs that are differentially expressed in patients with CRC. lncRNAs play important roles in gene regulation and affect many aspects of cellular homeostasis,^10^ and they are closely related to tumor occurrence and development.^11^

In this study, the first time this question has been addressed in northern China, we identified 1019 lncRNAs and 3221 mRNAs that are abnormally expressed in CRC tissues compared with adjacent nontumor tissues (|log2FoldChange|>1 and *q*‐value <0.05). Our results provide a reference for CRC diagnosis and treatment. Furthermore, our results demonstrated that lncRNA‐FIRRE‐201, SLCO4A1‐AS1‐202, LINC02163‐201, and FEZF1‐AS1‐203 were upregulated and SLC30A10‐201, PGM5‐AS1‐202, and ADIPOQ‐AS1‐201 were downregulated in CRC tissues compared with controls as assayed using qRT‐PCR. In addition, we demonstrated that mRNA‐NM_001062, NM_002994, and NM_001012964 were upregulated and NM_005182, NM_002594, and NM_001080400 downregulated in CRC tissues compared with controls, as assayed by qRT‐PCR. To verify its expression in the cell, we chose lncRNA‐FIRRE‐201 and SLCO4A1‐AS1‐202 for functional tests in CRC cells. Our results showed that knockdown reduced CRC cell proliferation, colony formation, migration, and invasion. The expression trends were consistent with the qRT‐PCR results. To improve the validation level, we selected only two lncRNAs (lncRNA‐FIRRE‐201 and SLCO4A1‐AS1‐202) from the 4 upregulated lncRNAs to perform functional experiments. The other two lncRNAs (LINC02163‐201 and FEZF1‐AS1‐203) also have the potential to serve as diagnostic markers for CRC, and further study is needed in the future.

We also observed that when the genes were clustered according to the expression pattern, the control group samples did not cluster together perfectly in the new lncRNAs compared with known lncRNAs. We concluded that there may be two reasons for this result. First, the reads of the expression are low, resulting in biased results. Second, there are errors due to low sample size.

Enrichment analysis also showed that these mRNAs are mainly associated with the components of extracellular matrix, suggesting that activation of the extracellular matrix plays a key role in the process of CRC. Many studies have found that a large number of factors involved in this process are produced in colorectal cancer tissue.^12^ The proliferation, differentiation, and invasion capabilities of tumor cells are regulated by various molecules. We also found that the mRNAs are mainly related to the chromosome, spindle, and centromeric regions, and thus mitosis. This suggests that the influence of these mRNAs on cell components might be related to mitosis. It is well known that mitosis is an important process for cell proliferation.^12^ Therefore, we conclude that these upregulated mRNA in colorectal cancer may make mitosis‐related cell components abnormal in result or function by different ways. In addition, cell cycle regulation is of great significance for maintaining the homeostasis of the body and preventing the occurrence of cancer. The abnormal regulation of cell cycle has been considered as an important intermediate link in tumor occurrence.^13^

We analyzed the first 10 mRNAs screened and found that in addition to the REG1B reported to be involved in the biological function of cancer cells, the remaining nine have been associated with CRC. Regenerating gene (REG) I plays key roles in cancer cell biology,^14^ which has not been reported for CRC but has for squamous esophageal cancer 14 and head and neck squamous cell carcinoma.^15^ There are reports that the REG family is composed of antiapoptotic factors and growth factors in the digestive system,^15^ and all REG family mRNAs are upregulated in inflammatory bowel disease.^16^ REG expression is higher in CRC, and REG therefore may be a potential marker. The expression of TCN1,^17^, ^18^ MMP7,^19^ and MAGE‐A3 20 was increased in CRC tissues. High CST1 expression enhances tumor metastasis and invasiveness in CRC.^21^ SLCO1B3 was validated, and its protein expression was closely related to expression of tumor proximal location and mismatch repair genes.^22^ KRT6B might be involved in the development, progression, and prognosis of human RCC.^23^ MMP1 is associated with the prognosis of patients with CRC, whereby patients with higher expression of these individual potential biomarkers exhibit poorer prognoses.^24^ COLL1A1 was identified as a crucial gene in protein‐protein interaction modules,^25^ and there is an association of ADIPOQ variants with CRC risk.^26^ The first 10 of mRNAs are expressed in the TCGA database, and only one—COLL1A1—is not found; the reasons may be race, territory, and sample size. So these DE genes, especially the top 10 mRNAs, are reproducible in larger cohort.

We were interested in finding differential genes from different samples that may be related to changes in cell pathways or gene functions. According to the results of pathway enrichment analyses and in combination with the number of enriched genes, the most potential networks for our differently expressed lncRNAs and mRNAs were in the cell cycle pathway, protein digestion and absorption pathway, cell adhesion molecules pathway, and alcoholism pathway. Some classical pathways, including Wnt signaling pathway, the PI3K‐Akt signaling pathway, the TGF‐β signaling pathway, the MAPK signaling pathway, and the p53 signaling pathway, are found in the CRC pathway diagram. This result was consistent with the conclusions of the accumulation studies. Recently, many reports have suggested that lncRNAs play important roles in CRC growth, migration, and proliferation.^27^, ^28^, ^29^, ^30^, ^31^ lnc‐PANDAR enhanced expression of E‐cadherin by inhibiting β‐catenin, N‐cadherin, Snail, vimentin, and Twisted expression, with effects on the epithelial‐mesenchymal transition (EMT).^27^ lnc‐NEAT1, as an oncogene, is of great significance for CRC progression. Knockdown of lnc‐NEAT1 in CRC cells not only causes apoptosis and growth arrest but also decreases Bcl‐2 and increases Bax expression by regulating the Akt signaling pathway.^28^ lnc‐TUG1 significantly contributes to CRC progression. Inhibition of TUG1 inhibited CRC cell proliferation, migration, and EMT.^29^ The AKT/mTOR signaling is a pivotal pathway participating in multiple physiological and pathological processes, including gene transcription, protein translation, cell cycle regulation, and proliferation.^30^ CRNDE is involved in the cell proliferation, migration, and invasion of CRC cells by increasing expression of TCF7L2 and activity of Wnt‐β‐catenin signaling through competitive binding to miR‐217.^31^ lncRNAs are crucial factors for tumor occurrence and development and require further study.

Our study has several limitations. We used next‐generation sequencing to study the differences in the expression profiles of lncRNAs and mRNAs in colorectal cancer tissues. Because the number was relatively small, it cannot be so precise to take into account the molecular classification, such as MSI, RAS, BRAF, and PIKCA. We did not measure the expression of identified tissue lncRNAs in serum samples to access the consistency of tissue and serum. We need more ROC curves to improve the diagnosis of CRC. Then, we can use Cox proportional hazards regression model analysis to explore the independent factors for the disease‐specific survival rate of patients with CRC. We evaluated lncRNA function in vitro; however, animal studies are required. It is unclear whether these lncRNAs are only specific for CRC. Thus, additional studies in other tumors are required. Furthermore, we should increase the number of clinical samples to confirm the function of the lncRNAs. We screened a large number of lncRNAs and mRNAs by sequencing techniques. Some of them were validated by RT‐PCR and partial cell function experiments. These lncRNAs and mRNAs may provide a basis for research and contribute to the development of novel therapeutics.

## CONFLICT OF INTEREST

The authors have declared that no conflict of interest exists.
