# Supplementary material for: Differentially expressed lncRNAs and mRNAs identified by NGS analysis in colorectal cancer patients
Source: Cancer Med. 2018 Jul 23;7(9):4650–64. doi: 10.1002/cam4.1696 (PMC6144144; doi:10.1002/cam4.1696)
Supplement: Supplementary file 1 [file CAM4-7-4650-s001.docx]

**Supplementary Table1**

| Primer name | sequnence |
| --- | --- |
| NM_001062f | CTCCCGAGCTTACATCTGACA |
| NM_001062r | AAAACTACATCCGCCTAAAACC |
| NM_001012964f | GCAGCAATCAGACTCAGCACC |
| NM_001012964r | TCTGCCTCCTCGTCTCAAAGC |
| NM_002594f | TCAGGCACGGTTTTCCAGTCT |
| NM_002594r | ATTTAATCACCTCTTTGGCTACGG |
| NM_002994f | ATTTTGGGATGAACTCCTTGC |
| NM_002994r | ACCACTATGAGCCTCCTGTCC |
| NM_005182f | AGGGTGACCAAGGAGCAAACT |
| NM_005182r | TTACCTCGGAGGACGATGAGA |
| NM_001080400f | GTCTGCTCCCCACCATTGTCT |
| NM_001080400r | GCCTCAGCACCTTCCAGAACT |
| FIRRE-201f | CCGAGTGAAGAGGATTGA |
| FIRRE-201r | GGAGAAGTGAAGCGAGGT |
| SLCO4A1-AS1-202f | ACACTTTCCAGCCTCTCAC |
| SLCO4A1-AS1-202r | CCGTCTGTTCCTGATTCTT |
| LINC02163-201f | AGACCAAGAACCCACAGGAAT |
| LINC02163-201r | GTTTAGAAGGCAGTGTACTGAATAG |
| FEZF1-AS1-203f | AAGAGGGGTGGAAAGGAAGA |
| FEZF1-AS1-203r | AAAACGAACTGGTGGCATTC |
| SLC30A10-201f | TCCCAAAAGGAGTCAACA |
| SLC30A10-201r | GCAGGGTGGCAATAATCT |
| PGM5-AS1-202f | AGGTTTCAACAGACGGCTTCA |
| PGM5-AS1-202r | AATTATGTATTGCCCCTCCCC |
| ADIPOQ-AS1-201f | TGCTAGAAATCAAACCCAGAG |
| ADIPOQ-AS1-201r | GGGAAACAGACACCTACCAAC |
| GAPDHf | CGGATTTGGTCGTATTGGG |
| GAPDHr | CTGGAAGATGGTGATGGGATT |
