# Supplementary material for: Differentially expressed lncRNAs and mRNAs identified by NGS analysis in colorectal cancer patients
Source: Cancer Med. 2018 Jul 23;7(9):4650–64. doi: 10.1002/cam4.1696 (PMC6144144; doi:10.1002/cam4.1696)
Supplement: Supplementary file 2 [file CAM4-7-4650-s002.docx]

**Supplementary Table2**

| Si-RNA | 5’-3’ |
| --- | --- |
| FIRRE-27 | GCC UAG GAC CUU UGU GGU ATT |
|  | UAC CAC AAA GGU CCU AGG CTT |
| FIRRE-358 | CCA GCU UUC UGA AGC AUA UTT |
|  | AUA UGC UUC AGA AAG CUG GTT |
| FIRRE-763 | CCA UGU ACA CCA UCA UCA ATT |
|  | UUG AUG AUG GUG UAC AUG GTT |
| SLCO4A1-992 | GCU UAG UGA CAU CCU GUA ATT |
|  | UUA CAG GAU GUC ACU AAG CTT |
| SLCO4A1-1139 | GGA ACA UUC AAA UGC UCU UTT |
|  | AAG AGC AUU UGA AUG UUC CTT |
| SLCO4A1-1355 | CCA UGA ACA UGG GUU CUU UTT |
|  | AAA GAA CCC AUG UUC AUG GTT |
| NC | UUC UCC GAA CGU GUC ACG UTT |
|  | ACG UGA CAC GUU CGG AGA ATT |

In this paper, si-FIRRE-201-1 is Firre-27, Firre-358, Firre-763 mixture, and si-FIRRE-201-2 is Firre-27. Si-SLCO4A1-AS1-202-1 is SLCO4A1-992, SLCO4A1-1139, SLCO4A1-1355 mixture, and si- SLCO4A1-AS1-202-2 is SLCO4A1-992.
